# Targeted and cellular therapies in lymphoma: Mechanisms of escape and innovative strategies

**DOI:** 10.3389/fonc.2022.948513

**Published:** 2022-09-12

**Authors:** Anagha Deshpande, Javier Munoz

**Affiliations:** ^1^ Mayo Clinic Alix School of Medicine, Scottsdale, AZ, United States; ^2^ Division of Hematology and Oncology, Mayo Clinic, Phoenix, AZ, United States

**Keywords:** lymphoma, targeted therapy, resistance, mechanism of action, CAR T-cells, tazemetostat, cerdulatinib

## Abstract

The therapeutic landscape for lymphomas is quite diverse and includes active surveillance, chemotherapy, immunotherapy, radiation therapy, and even stem cell transplant. Advances in the field have led to the development of targeted therapies, agents that specifically act against a specific component within the critical molecular pathway involved in tumorigenesis. There are currently numerous targeted therapies that are currently Food and Drug Administration (FDA) approved to treat certain lymphoproliferative disorders. Of many, some of the targeted agents include rituximab, brentuximab vedotin, polatuzumab vedotin, nivolumab, pembrolizumab, mogamulizumab, vemurafenib, crizotinib, ibrutinib, cerdulatinib, idelalisib, copanlisib, venetoclax, tazemetostat, and chimeric antigen receptor (CAR) T-cells. Although these agents have shown strong efficacy in treating lymphoproliferative disorders, the complex biology of the tumors have allowed for the malignant cells to develop various mechanisms of resistance to the targeted therapies. Some of the mechanisms of resistance include downregulation of the target, antigen escape, increased PD-L1 expression and T-cell exhaustion, mutations altering the signaling pathway, and agent binding site mutations. In this manuscript, we discuss and highlight the mechanism of action of the above listed agents as well as the different mechanisms of resistance to these agents as seen in lymphoproliferative disorders.

## Introduction

Lymphomas are a group of malignancies characterized by the uncontrolled proliferation of either mature B-lymphocytes or T-lymphocytes. Lymphomas can be further classified as Hodgkin or non-Hodgkin based on the presence or absence of Reed-Sternberg cells, respectively. The treatment for lymphomas includes active surveillance, chemotherapy, immunotherapy, radiation therapy, and even stem cell transplant. In terms of chemotherapy, for Hodgkin lymphoma (HL), the front-line therapy has been ABVD (doxorubicin, bleomycin, vinblastine, dacarbazine), but despite great overall survival rates, around 30-40% of patients relapse within the first two years after treatment (1). On the other hand, for non-Hodgkin lymphoma (NHL), R-CHOP (rituximab, cyclophosphamide, doxorubicin, vincristine, and prednisone) has been the front-line therapy, and despite complete responses, around 40-50% of patients develop refractory/relapsed (R/R) disease (2). It should be noted that it took decades of trial and error of trying to improve upon the backbone of ABVD and CHOP to eventually develop the brentuximab vedotin + AVD (3) and rituximab + CHOP treatment regimens that are now part of the first-line therapies. One such example of the trial-and-error process is that after a pivotal trial found unacceptable levels of pulmonary toxicity with brentuximab vedotin + ABVD, bleomycin was removed from the regimen (4). In addition, after many trials, polatuzumab vedotin was also found to significantly improve the R-CHP treatment regimen in the first-line treatment setting for NHLs (5, 6). To address high R/R rates, technological advances have led to the development of targeted therapies against driver molecular aberrations that have emerged as highly effective treatment options in patients whose malignancies harbor the allotted target (7–9). A targeted therapy can be defined as an agent that targets a critical molecular pathway involved in tumorigenesis (10). Furthermore, advances facilitating rapid genomic profiling have allowed for the formation of hypotheses regarding which patients may benefit more from a targeted therapy based on their genetic subtype (11). However, many cancers have strategically developed means to outsmart the highly precise medicines to confer resistance. Thus, we will discuss the mechanisms of escape to various targeted therapies noted in lymphoproliferative disorders.

## Targeted therapies

### Rituximab

A chimeric monoclonal antibody, rituximab targets the CD20 antigen expressed on lymphocytes and induces cell lysis upon binding antibody-dependent cellular cytotoxicity (ADCC) and complement-dependent cytotoxicity (CDC). Rituximab is used in a variety of disorders, and when it comes to malignancies, it is mainly used to treat NHLs and chronic lymphocytic leukemia. Currently, the FDA has approved rituximab for use in treating NHLs, chronic lymphocytic leukemia (CLL), rheumatoid arthritis, granulomatosis with polyangiitis, and microscopic polyangiitis. Mechanisms of resistance to rituximab are not completely understood, since the therapy relies on the host immune system to mount an immune response, and thus, host factors can also significantly impact efficacy (12). However, three main mechanisms are postulated. The first is that tumor cells have developed ways to block CDC; analysis of rituximab-resistant cell lines has shown that these cells express high levels of membrane complement regulatory proteins (mCRP) – such as CD46, CD55, and CD59 – and these inhibitory proteins block the activation of the complement cascade (13). This theory has been supported by preclinical studies displaying that neutralizing mCRPs with antibodies lead to increased effectiveness of rituximab (14). Next, though rituximab inhibits B-cell lymphoma 2 (Bcl-2) expression to promote cell apoptosis, a study found that prolonged exposure to rituximab led to the downregulation of pro-apoptotic proteins Bcl-2 antagonist/killer (BAK) and Bcl-2 associated X (BAX), conferring resistance (15). Finally, the most supported mechanism of resistance is downregulation of CD20, the target antigen. Studies have identified C-terminal deletions in the CD20 gene as well as decreased expression of CD20 mRNA in cells found to be CD20 negative after rituximab exposure (16, 17). Since rituximab is utilized throughout many phases of lymphoma treatment – first-line, maintenance, and salvage – studies are being conducted to develop strategies for navigating these mechanisms of resistance.

### Brentuximab vedotin

A chimeric antibody-drug conjugate, brentuximab vedotin targets the CD30 antigen expressed on lymphocytes to trigger cell death. It has been FDA-approved to treat classical HL and systemic anaplastic large cell lymphoma. Additionally, brentuximab vedotin is a part of the front-line therapy for HL and T-cell NHL (18, 19). CD30 is expressed on activated lymphocytes thus it is an attractive target for therapies (20). Upon binding, the drug is internalized into the cell and subsequently releases the potent microtubule inhibitor monomethyl auristatin E (MMAE) to inhibit cell differentiation and induce apoptosis (20). An *in vitro* study analyzing brentuximab vedotin resistant cells found that CD30 expression was not significantly lowered in these cells (21). Instead, the resistant cells upregulated the expression of the multi-drug resistance (MDR1) gene and its subsequent product, P-glycoprotein, to confer resistance (21). Additionally, the cells displayed decreased intracellular accumulation of MMAE and increased efflux of MMAE, allowing the cells to avoid death (21). This mechanism of resistance has been further supported by a phase 1 study evaluating the effects of two broad multi-drug resistance modifiers, cyclosporine A and verapamil, on brentuximab vedotin resistance in patients with brentuximab vedotin-resistant HL (22). This study found that inhibiting *MDR1* restored sensitivity to brentuximab vedotin, increased intracellular MMAE levels, and improved overall brentuximab vedotin activity (22).

### Polatuzumab vedotin

Polatuzumab vedotin is another antibody-drug conjugate that targets the CD79b antigen expressed on lymphocytes, and it has been FDA-approved for treating R/R diffuse large B-cell lymphoma (DLBCL). When antigens bind to the B-cell receptor (BCR), the ligand-receptor complex gets internalized into the cell so that the antigen can be presented on major histocompatibility complex (MHC) class II molecules on the B-cell surface. This process relies heavily on the proper functioning of CD79, a heterodimer of CD79a and CD79b, and within this, CD79b is the dominant player (23). Since CD79b is expressed on most cells of B-cell lymphomas and leukemias, it serves as a prime target for therapies such as polatuzumab vedotin (23). Upon binding to CD79b, polatuzumab vedotin induces cell death in a similar manner to brentuximab vedotin (23). Though polatuzumab vedotin is currently approved (in combination with bendamustine plus rituximab) for use in the R/R setting for DLBCL (24), the POLARIX study found that among 879 patients, the risk of disease progression, relapse, or death was lower in the group treated with polatuzumab vedotin + R-CHP when compared to the group treated with standard R-CHOP – highlighting that polatuzumab vedotin + R-CHP may soon emerge as a part of first-line therapy for DLBCL (5). POLARIX was a confirmatory phase 3 trial based on the positive toxicity profile seen regarding the use of Polatuzumab in the phase 1-2b study in patients with previously untreated DLBCL (25). Utilizing flow cytometry to analyze CD79b cell-surface expression, one study identified that a minimal threshold of 6.82 geometric mean fluorescence intensity units for CD79b expression must be present for anti-CD79b ADCC to be effective (25). Therefore, the primary mechanism of resistance to polatuzumab vedotin is downregulation of CD79b expression (25). However, the ROMULUS phase 2 clinical trial identified resistance to MMAE as another mechanism in patients with R/R diffuse large B-cell lymphoma (DLBCL) and follicular lymphoma (FL) (26). This trial compared polatuzumab vedotin to pinatuzumab vedotin – another antibody-drug conjugate targeted at CD22 (26). In this trial, six patients who had no response originally switched to the other antibody-drug conjugate, and none of these patients responded to the other drug – highlighting that their malignancies had developed resistance to MMAE, not the respective CD drug targets (26). Strategies to overcome resistance to polatuzumab vedotin are being devised and studied (27), and indeed many combinatorial approaches are under development in clinical trials including PolaR-ICE (rituximab, ifosfamide, carboplatin, and etoposide) (NCT04665765), polatuzumab vedotin + GemOX (gemcitabine and oxaliplatin) (NCT04182204), and polatuzumab vedotin + mosenutuzumab (NCT03671018) (28).

### Nivolumab

Nivolumab is a monoclonal antibody that binds to and blocks the programmed death receptor-1 (PD-1). It has been FDA-approved to treat classical Hodgkin lymphoma (cHL), melanoma, non-small cell lung cancer, malignant pleural mesothelioma, renal cell carcinoma, squamous cell carcinoma of the head and neck, urothelial carcinoma, colorectal cancer, hepatocellular carcinoma, esophageal cancer, and gastric cancer. Also known as CD279, PD-1 is a checkpoint protein on T-cells and B-cells that binds to programmed death-ligand 1 and 2 (PD-L1 and PD-L2) on other cells of the body to prevent immune cells from attacking other cells in the body (29). Malignant cells express high levels of PD-L1 to help shield them from an immune system response (29). Additionally, the genes for PD-L1 and PD-L2 are located on chromosome 9p24.1, and amplification of 9p24.1 was found to be associated with increased expression of PD-L1 in HL (30). Therefore, blocking the interaction between PD-1 and PD-L1 enhances the immune system’s anti-tumor response and delays tumor growth (29). Mechanisms of resistance to immune checkpoint inhibition involve inadequate T-cell attraction and activation in addition to impaired T-cell effector functions. In cHL, the Hodgkin Reed-Sternberg (HRS) cells produce vascular endothelial growth factor (VEGF) which induces regulatory T-cell proliferation and increases the expression of inhibitory receptors, including PD-1 (31). This, in turn, leads to T-cell exhaustion (31). Thus, a tumor microenvironment with a higher proportion of regulatory T-cells and inhibitory receptors can alter the efficiency of PD-1 blockade therapy (31). Next, tumor cells can have absent or aberrant HLA expression which compromises antigen presentation and affects immune checkpoint inhibition efficacy (32). In fact, in around 70% of cHL cases, HLA class I surface expression is lost (32). Tumor cells can resist PD-1 blockade therapy by increasing the production of indoleamine 2,3-dioxygenase (IDO), the initial and rate-limiting enzyme involved in the degradation of tryptophan (33). Finally, HRS cells have decreased levels of adenosine deaminase, the enzyme involved degrading the purine adenosine (34). This increases levels of adenosine in cHL cells which activates the alternative degradation pathway involving CD32, CD203a, and CD73 (35). However, increased CD73 expression has been shown to directly reduce the effectiveness of PD-1 blockade therapy (36). Thus, increased adenosine levels in cHL cells confers resistance to immune checkpoint inhibitors such as nivolumab. Strategies to overcome resistance to nivolumab are currently being developed and studied (37), and certainly many combinatorial approaches are under development in clinical trial including nivolumab + AVD (NCT03907488) and brentuximab + nivolumab with or without ipilimumab (NCT01896999) (38).

### Pembrolizumab

Like nivolumab, pembrolizumab is another monoclonal antibody that binds to and blocks PD-L1 (39). It has been FDA-approved for cHL, primary mediastinal large B-cell lymphoma, melanoma, non-small cell lung cancer, small cell lung cancer, head and neck squamous cell cancer, urothelial carcinoma, colorectal cancer, gastric cancer, esophageal cancer, cervical cancer, hepatocellular carcinoma, Merkel cell carcinoma, renal cell carcinoma, endometrial carcinoma, cutaneous squamous cell carcinoma, and triple-negative breast cancer. Mechanisms of resistance to pembrolizumab are similar to those outlined for nivolumab. Currently, studies have identified ways to circumvent resistance to immune checkpoint inhibition in solid tumors (40, 41); however, studies are being conducted to develop strategies to overcome resistance in lymphoproliferative disorders (42, 43).

### Mogamulizumab

A monoclonal antibody, mogamulizumab targets the C-C chemokine receptor 4 (CCR4) to inhibit this signal transduction pathway. This, subsequently, prevents the chemokine-mediated migration and proliferation of T-cells (44). Since CCR4 is expressed on almost all T-cells in cutaneous or peripheral T-cell lymphomas or leukemias, mogamulizumab has emerged as an attractive therapeutic option (44). It is currently FDA-approved for use in treating R/R mycosis fungoides and Sézary syndrome. A study on 19 patients with either mycosis fungoides or Sézary syndrome found that though all patients had T-cells with CCR4 expression prior to starting treatment, all of them had to discontinue mogamulizumab due to lack or loss of response to therapy (45). After stopping treatment, in 57% of patients, CCR4 expression was no longer detected by immunohistochemistry (45). Targeted DNA-sequencing of these samples found that loss of CCR4 expression occurred both with and without genomic alterations in the *CCR4* gene (45). Additionally, the study identified that none of the patients that experienced a loss of CCR4 expression benefitted from a second course of mogamulizumab (45). It should also be noted that this study also identified a subset of patients with high CCR4 expression and an undetermined mechanism of resistance to mogamulizumab (45). Further investigations are currently underway to better understand these mechanisms of resistance and devise strategies to overcome them. Many combinatorial approaches are under development in clinical trials including mogamulizumab plus magrolimab (NCT04541017) and mogamulizumab plus natural killer cells (NCT04848064) (46).

### Vemurafenib

A small-molecule kinase inhibitor, vemurafenib inhibits the BRAF serine/threonine protein kinase with the V600E or V600K mutation. Cells with this aberrant molecule have unregulated cell growth through the mitogen activated protein kinase (MAPK) pathway (47, 48). Thus, this targeted therapy has been effectively used for treating melanoma and hairy cell leukemia (HCL). However, it is currently FDA-approved for unresectable or metastatic melanoma with the BRAF V600E mutation as detected by an FDA-approved test. Furthermore, it is also FDA-approved for patients with Erdheim-Chester disease, which is a rare histiocytic disorder (49, 50), that carry the BRAF V600E mutation (51). Initial studies on vemurafenib in HCL found a missense mutation in insulin receptor substrate 1 (IRS1) in addition to the BRAF V600E mutation that induced the MAPK pathway through activation of extracellular signal-regulated kinase (ERK) (52). By allowing BRAF to be bypassed to activate ERK, the *IRS1* mutation conferred resistance to treatment (52). Additionally, the study identified a mutation in the Kirsten rat sarcoma (*KRAS*) gene, a protein involved in the RAS/MAPK pathway, that also mediated resistance to vemurafenib (52). Thus, this study outlined both ERK dependent and independent mechanisms of resistance to vemurafenib in HCL (52). Another study also found *IRS1* and *KRAS* mutations in vemurafenib-resistant HCL cell lines, however, this study also found loss-of-function mutations in the neurofibromatosis 1 and 2 genes (*NF1* and *NF2*) which contributed to the lack of response (53). To address these mechanisms of resistance, a study was conducted on a 74-year-old patient with vemurafenib-resistant HCL with many resistance-conferring mutations (including *KRAS*) present in the cell lines (54). MEK is a protein kinase upstream of ERK in the MAPK pathway, and with the addition of the MEK inhibitor cobimetinib to vemurafenib, the patient responded to such combination therapy (54). The bone marrow showed suppression of ERK activity (54). At 12 month follow up, the patient showed continued response and remained asymptomatic – highlighting MEK inhibition as a potential option for navigating resistance to vemurafenib (54).

### Crizotinib

A tyrosine kinase receptor inhibitor, crizotinib specifically targets anaplastic lymphoma kinase (ALK), hepatocyte growth factor receptor (HGFR, c-MET), and Recepteur d’Origine Nantais (RON). It is currently FDA-approved for treating metastatic non-small cell lung cancer with ALK or ROS-1 positivity per an FDA-approved test, ALK-positive anaplastic large cell lymphoma, and ALK-positive inflammatory myofibroblastic tumors. In non-small cell lung cancer, studies found a chromosomal rearrangement creating a gene fusion product that resulted in a constitutively active ALK protein as the oncologic driver (55). Other studies on anaplastic large cell lymphoma, the most common T-cell NHL in children, found that tumor progression was primarily driven by a fusion product between *ALK* and mainly nucleophosmin 1 (NPM1) called NPM-ALK (56). For these reasons, crizotinib has emerged as an effective therapy for these malignancies. In cases where crizotinib resistance developed, studies identified *ALK* mutations conferring resistance; however, studies are being conducted to see if this can be overcome by using newer-generation ALK inhibitors such as alectinib, ceritinib, brigatinib, and lorlatinib (57). In other cases where crizotinib resistance developed and *ALK* mutations were not identified, a study used genome-wide clustered regularly interspaced short palindromic repeats (CRISPR) analysis to look for overexpressed genes that could be conferring resistance (58). This study found that in around 30% of crizotinib-resistant cell lines, the *IL10RA* gene for the IL-10 signaling pathway was overexpressed in cells both with and without *ALK* mutations (58). Through further investigation, this study identified how the IL-10 pathway ultimately activated signal transducer and activator of transcription 3 (STAT3), a molecule that promotes cell survival (58). Furthermore, STAT3 was found to bind to the promoter for *IL10RA* and upregulate its expression – ultimately creating a feedback loop that bypasses NPM-ALK and promotes cell survival through increased STAT3 activity (58). However, the authors of this study did note that further investigation of this mechanism of resistance is needed (58). Strategies to overcome resistance have been identified in non-small cell lung cancer, but studies are needed to develop these strategies in lymphoproliferative disorders (59).

### Ibrutinib

Constitutive B-cell receptor signaling pathway activation has been implicated in numerous B-cell malignancies. One enzyme in this pathway is Bruton tyrosine kinase (BTK), and this enzyme plays a crucial role in modulating cytokine and integrin expression for B-cell trafficking and proliferation (60). Thus, ibrutinib was developed to specifically inhibit BTK (although other enzymes are indirectly affected too) and provide a therapeutic effect in malignancies such as CLL, mantle cell lymphoma (MCL), DLBCL, and Waldenström’s macroglobulinemia (WM) (60). It is currently FDA-approved for use in treating MCL, CLL, small lymphocytic lymphoma (SLL), WM, MZL, and chronic graft versus host disease. For understanding mechanisms of resistance, early studies utilized whole-exome sequencing to compare baseline and relapse genomes of patients with CLL who had been treated with ibrutinib (61). One study concluded that resistance developed due to the BTK^C481S^ mutation in the binding site on BTK for ibrutinib (61). This study also identified a mutation in the 1-phosphatidylinositol-4,5-bisphosphate phosphodiesterase gamma-2 enzyme (PLCG2) – an enzyme that is further downstream of BTK in the B-cell signaling pathway; however its implication in resistance development was not entirely clear (61). Further investigations identified that this mutation did in fact contribute to ibrutinib-resistance in both CLL and WM (62, 63). It should be noted that within each malignancy, other genetic mutations have been identified conferring resistance to ibrutinib; however, the *BTK* and *PLCG2* mutations are the most common in patients with CLL. *BTK* and *PLCG2* mutations conferring resistance to ibrutinib have also been documented in MZL (64). In MCL specifically, studies have identified sustained distal B-cell receptor signaling pathway activation through the classical and alternative NFkB pathways as a mechanism underlying primary resistance to ibrutinib (65). In WM, *BTK* and PLCG2 mutations have been identified as mechanisms of resistance (66), however, responses to ibrutinib are also highly dependent on whether patients have the CXCR4^WHIM^ mutation that confers resistance to ibrutinib (67, 68). **Table 1** summarizes the mutated genes that lead to resistance to ibrutinib resistance in lymphoproliferative disorders. In terms of molecular changes that contribute to resistance, studies have found that resistant DLBCL lines overexpress CD79B while resistant MCL lines overexpress MYC (70, 71). Increased expression of XPO1 and loss of TRAIL-induced apoptosis has been identified as a mechanism of resistance to ibrutinib in CLL (84, 85), while deletions on chromosomes 6q and 8p have been identified in WM (86, 87). Hence, not every lymphoproliferative disorder will display the same mechanism of resistance when exposed to BTK inhibitors. Resistance to ibrutinib has been overcome through the development of second-generation BTK inhibitors that target BTK with much more specificity compared to ibrutinib. **Table 1** also summarizes therapeutical strategies to circumvent resistance.

**Table 1:** Summary table of the mechanisms of resistance to targeted therapies in lymphoproliferative disorders.

Agent	Target	Primary clinical indications	FDA-approval	Mechanism of resistance
Rituximab	CD20	NHLs, CLL	NHLs, CLL, rheumatoid arthritis, granulomatosis with polyangiitis, and microscopic polyangiitis	Expression of inhibitory proteins that block complement activation (13)
				Downregulation of BAK and BAX (15)
				Downregulation of CD20 (16, 17)
Brentuximab vedotin	CD30	cHL, anaplastic large cell lymphoma	cHL and systemic anaplastic large cell lymphoma	Increased expression of *MDR1* and P-glycoprotein (21)
Polatuzumab vedotin	CD79b	DLBCL	R/R DLBCL	Downregulation of CD79b expression (25)
				Resistance to MMAE (26)
Nivolumab	PD-1	cHL	cHL, melanoma, non-small cell lung cancer, malignant pleural mesothelioma, renal cell carcinoma, squamous cell carcinoma of the head and neck, urothelial carcinoma, colorectal cancer, hepatocellular carcinoma, esophageal cancer, and gastric cancer	Altered tumor microenvironment with increased regulatory T-cells and inhibitory receptors (31)
				Absent or aberrant HLA expression (32)
				Increased IDO production (33)
				Increased levels of adenosine that increases CD73 expression (34, 36)
Pembrolizumab	PD-1	cHL, B-cell lymphoma	cHL, primary mediastinal large B-cell lymphoma, melanoma, non-small cell lung cancer, small cell lung cancer, head and neck squamous cell cancer, urothelial carcinoma, colorectal cancer, gastric cancer, esophageal cancer, cervical cancer, hepatocellular carcinoma, Merkel cell carcinoma, renal cell carcinoma, endometrial carcinoma, cutaneous squamous cell carcinoma, and triple-negative breast cancer	Same mechanisms above as nivolumab
Mogamulizumab	CCR4	Cutaneous T-cell lymphoma	R/R mycosis fungoides and Sézary syndrome	Loss of CCR4 expression (45)
Vemurafenib	BRAF	Hairy cell leukemia	Unresectable or metastatic melanoma with the BRAF V600E mutation, Erdheim-Chester disease	*IRS1* mutation (52)
				*KRAS* mutation (52)
				Loss of function mutations in *NF1* and *NF2 (* 53)
Crizotinib	ALK	Anaplastic large cell lymphoma	Metastatic non-small cell lung cancer with ALK or ROS-1 positivity, ALK positive anaplastic large cell lymphoma, ALK positive inflammatory myofibroblastic tumor	*ALK* mutation (57)
				Overexpression of *IL10RA* in the IL-10 signaling pathway (58)
				Increased STAT3 activity (58)
Ibrutinib	BTK	CLL	MCL, CLL, SLL, WM, MZL, and chronic graft versus host disease.	BTK^C481S^ mutation (61)
				PLCG2 enzyme mutation (69)
				Overexpression of CD79B (70)
				Overexpression of MYC (71)
Cerdulatinib	JAK-STAT	T-cell lymphoma	Orphan drug designation for peripheral T-cell lymphoma	Generation of *MYB-TYK2* fusion gene (72)
				Hyperactivity of JAK-STAT signaling pathway (72)
				*EP300* mutation (73)
Idelalisib	PI3K	CLL	Approved January 2014; Withdrawn January 2022	Increased IGF1R expression (74)
				*KRAS*, *BRAF*, and *MAP2K1* mutations (75)
Copanlisib	PI3K	FL	Relapsed FL	Upregulation of IL-6 to induce STAT3 and STAT5 pathways (76)
				Downregulation of genes involved in cell adhesion, antigen presentation, and interferon response (77)
				Upregulation of cytokine, NF-KB, MAPK, and JAK-STAT pathways and negative regulators of apoptosis (77)
Venetoclax	Bcl2	CLL, SLL	CLL, SLL, and AML	G101V and D103Y mutations in Bcl2 (78)
				*BTG1* and *BRAF* mutations (79)
				*CDKN2A/B* deletions (79)
				Amplification of PD-L1 expression (79)
Tazemetostat	EZH2	FL	Epithelioid sarcomas and R/R FL	Increased activation of IGF1R and MEK, PI3K pathways (80)
				Acquired mutations in *EZH2* altering drug binding (80)
CAR T-cells	CD19, CD20	DLBCL, FL, MCL	DLBCL, FL, MCL, B-cell ALL, and multiple myeloma (please refer to **Table 3** for further breakdown)	Nonsense mutation mediated CD19 decay (81)
				Downregulation of CD20 expression (69)
				B-cell lineage switching from lymphoid to myeloid through *MLL (* 82)
				Increased PD-L1 signaling leading to T-cell exhaustion (83)

### Cerdulatinib

In the pathogenesis of B-cell malignancies, the Janus kinase and Signal Transducer and Activator of Transcription (JAK-STAT) pathway produces an active STAT3 molecule that promotes cell survival even in a hostile tumor microenvironment (88). Studies showed that inactivating either JAK or STAT3 decreased cell proliferation and increased apoptosis, and this provided the rationale for developing cerdulatinib – a small-molecule ATP-competitive inhibitor of SYK, JAK1, JAK2, JAK3, and TYK2 (88). This therapy has FDA orphan drug designation for treating peripheral T-cell lymphoma. It should be noted that studies have highlighted that cerdulatinib can overcome ibrutinib-resistance in R/R CLL (89). Preliminary data has also shown the drug’s efficacy in treating small lymphoplasmacytic lymphoma (SLL), FL, DLBCL, ALL, and peripheral T-cell lymphoma (PTCL). *In vitro* studies have modeled several mechanisms of resistance to cerdulatinib in ALL (72). The first was that long-term exposure to the drug facilitated the generation of the *MYB-TYK2* fusion gene that conferred resistance (72). Next, resistant cells with the *MYB-TYK2* fusion protein displayed hyperactivation of the JAK/STAT signaling pathway, leading to no response to the drug (72). However, withdrawing the drug for a brief period did re-sensitize the cells to treatment (72). In a phase 1 trial, eight patients with R/R CLL were given cerdulatinib, and two patients were found to have disease progression with treatment (73). These patients were found to have mutations in *BTK*, *TP53*, and *EP300*. Furthermore, it was proposed that the mutation in *EP300*, a gene encoding a histone acetyltransferase, was the mostly likely mechanism of resistance of cerdulatinib (73). Strategies to overcome resistance to cerdulatinib are highly awaited. For example, there was a phase 2 trial combining cerdulatinib with or without rituximab in patients with lymphoma (73).

### Idelalisib

In many cancers, the phosphoinositide 3-kinase (PI3K) signal transduction pathway is highly active, which is why developing agents targeting PI3K was previously attractive (90). However, a challenge that arises is that four distinct PI3K isoforms exist with partially overlapping functions and differing toxic effects (90). One such agent is idelalisib, a selective inhibitor of the delta isoform of PI3K, which has shown strong efficacy in treating B-cell malignancies with an acceptable side-effect profile (90). This drug was previously FDA-approved for treating CLL, FL, and SLL (90). However, there was a voluntary withdrawal of the indication for SLL and FL in 2022 (91). **Table 2** illustrates select PI3K inhibitors, their clinical indications, and FDA-approval status. *In vitro* studies evaluating idelalisib resistance in CLL found that it is associated with increased expression of the insulin-like growth factor 1 receptor (IGF1R) (74). Furthermore, this study also found that cells became re-sensitized to treatment when an IGF1R inhibitor was utilized (74). Another study found that CLL cells became resistant to idelalisib with increased and constitutive MAPK pathway activation, and this allowed for communication between the PI3K and MAPK pathways that circumvented PI3K inhibition (75). This study also identified that increased MAPK pathway activation was associated with the acquisition of mutations in *KRAS, BRAF*, and *MAP2K1 (* 75).

**Table 2:** Summary of mechanisms of resistance to ibrutinib in lymphoproliferative disorders and strategies to overcome resistance.

Mutated gene/Aberration	Mechanism of resistance	Conditions					Possible treatment strategy	References
		CLL	MCL	MZL	DLBCL	WM		
*BTK* (covalent)	Reversible ibrutinib binding	+	+	+		+	Third generation BTK inhibitors, PROTAC-BTK, inhibitors of LYN and SYK	(61–124)
*PLCG2*	BTK-independent activation	+	+	+		+	Inhibitors of RAC2, LYN, and SYK	(61–120, 125, 126)
*CARD11*	Increased NFkB signaling	+	+		+	+	Proteasome or MALT1 inhibitor	(63, 127–129)
*BIRC3, TRAF2, TRAF3*	Increased NFkB signaling		+				MP3K14 inhibitor	(130, 131)
*CCND1*	Cell cycle progression		+					(132)
*CCDKN2A*	Cell cycle progression		+				PRMT5 inhibitor	(133)
*TNFAIP3*	Increased NFkB signaling				+			(129)
*KLHL14*	Increased MYD88-TLR9-BCR super-complex signaling				+		Inhibition of BCR-dependent NFkB activation/mTOR inhibitors	(134)

+: Some pre-clinical or clinical evidence available that this particular pathway may play a role regarding resistance at the time of publication. Possible treatment strategies to overcome resistance are mainly theoretical based on pre-clinical hypotheses. The intention for this table is to show that the mechanisms of resistance may differ among lymphoproliferative disorders. This table is not meant to be comprehensive as there may be more mechanisms of resistance and more possible treatment strategies to overcome resistance involved in a particular pathway for any of these conditions particularly as our knowledge evolves over time.

*Non-genetic mechanisms of resistance to ibrutinib in lymphoproliferative disorders include PI3K-Akt pathway activation (which can possibly be overcome by PI3K, mTOR, or XPO1 inhibitors) (84, 135–143), JAK-STAT pathway activation (which can possibly be overcome by dual SYK/JAK-STAT inhibitors) (89), MYC activation (which can possibly be overcome by an HSP90 inhibitor) (71), MAPK pathway activation (which can possibly be overcome by an MEK inhibitor) (144, 145), BCL2 activation (which can possibly be overcome by an BCL2 inhibitor) (146–149), metabolic reprogramming (which can possibly be overcome by an oxidative phosphorylation inhibitor) (133, 150), integrin-mediated protection (which can possibly be overcome by VLA4 inhibition) (151, 152), and resistant cancer stem cells (which can possibly be overcome by an Wnt pathway inhibitor) (153).

### Copanlisib

Similar to idelalisib, copanlisib is a highly selective and potent intravenous PI3K inhibitor, yet it is unique because it can target multiple isoforms of PI3K, making it a pan-PI3K inhibitor (92, 93). For example, its unique affinity to the alpha isoform of PI3K (which is present in the pancreas) explains some of its toxicities including hyperglycemia (94). Furthermore, the intravenous route of administration as well as intermittent dosing schedule of the drug have been suggested to portray a more favorable tolerability profile compared to oral PI3K inhibitors (95). Nevertheless, the intravenous administration can also be cumbersome for patients that live far from a cancer center. It has been FDA-approved for relapsed FL. For mechanisms of resistance, a study on B-cell lymphoma resistant cells identified upregulation of IL-6, and IL-6 was able to independently activate STAT3 or STAT5 pathways to confer resistance to PI3K inhibition (76), thus the STAT pathway may be a relevant mechanism of resistance for some lymphoproliferative disorders (96). In resistant MZL cells, gene expression profiling showed upregulation of cytokine, NF-KB, MAPK, and JAK-STAT signaling pathways as well as the negative regulators of apoptosis (77), CD44 and JUN, as a mechanism underlying resistance (77). Furthermore, the cells showed decreased expression of genes involved in cell adhesion (ITGA4, ITGB1), antigen presentation, and interferon response (PARP12, GBP6) (77). This study also used flow cytometry to identify increased CXCR4 surface expression on resistant cells, and subsequently, the addition of a CXCR4 inhibitor overcame resistance to copanlisib (77).

### Venetoclax

Venetoclax is an inhibitor of B-cell lymphoma 2 (Bcl2), a pro-survival molecule that regulates the intrinsic apoptosis pathway. This drug is currently FDA-approved to treat CLL, SLL, and acute myeloid leukemia (AML). By binding to Bcl2, venetoclax enables the Bim and BH3 proteins to activate the pro-apoptotic molecules, Bax and Bak. Activation of these molecules commits the cell to apoptosis through the intrinsic mitochondrial pathway and prohibits further cell proliferation. However, malignant cells have developed many mechanisms of resistance to the drug. Some studies have identified mutations in the BH3 binding groove of Bcl2 that led to a protein conformation change hindering the ability of venetoclax to bind to Bcl2 and ultimately conferring resistance (97). Additionally, G101V and D103Y mutations in Bcl2 were identified which also interfere with the drug binding to Bcl2 (78). Other studies looking at patients with R/R CLL identified many genetics aberrations in cancer-related genes that conferred resistance to treatment. These included: mutations of *BTG1* and *BRAF*, deletions in *CDKN2A/B*, and amplification of PD-L1 expression – suggesting multiple mechanisms of resistance (79). Combination treatment strategies have been developed to improve the clinical efficacy and studies have shown improved response rates with venetoclax in combination with various agents including cytarabine, ibrutinib, rituximab, or bendamustine (98). Additional studies are currently being conducted to develop optimal combination regimens (98).

### Tazemetostat

Enhancer of zeste homolog 2 (EZH2) is a part of the polycomb group gene (PcG) family, and this is a group of epigenetic regulators that represses transcription (99). Aberrant EZH2 expression and signaling has been implicated in the pathogenesis of various cancers, which led to the development of the EZH2 inhibitor, tazemetostat (99). This agent is FDA-approved to treat epithelioid sarcomas and R/R FL. Although the agent is FDA approved for FL, we are still trying to elucidate the mechanisms of resistance to tazemetostat in FL (80, 100). For example, it has been described that resistance to EZH2 inhibitors in DLBCL occurs due to the activation of survival pathways and acquired EZH2 mutations that prevent drug binding (80). Resistant DLBCL cells have been found to display increased activation of IGF1R as well as the MEK and PI3K pathways, conferring resistance to EZH2 inhibition (80). Additionally, this study identified acquired mutations in the gene for EZH2 that included EZH2^Y641F^, EZH2^C663Y^, EZH2^E720G^, and EZH2^Y726F^ (80). These mutations prevented drug binding to the EZH2 mutants which decreased the effectiveness of treatment (80). Strategies to overcome resistance to tazemetostat are highly awaited in lymphoproliferative disorders.

### CAR T-cells

CAR T-cell therapy has emerged as the breakthrough treatment for numerous hematological malignancies. The basic principle behind this autologous therapy is genetically engineering and modifying a person’s T-cells to display a tumor antigen-binding receptor that directs the T-cells to mount a response against tumor cells (101). A CAR construct is a genetically engineered antigen receptor that binds to a target antigen (101). The CAR construct, of the 3 FDA approved constructs currently in the market for lymphoma, is made to target cluster of differentiation (CD) molecules that are expressed on malignant cells (101). For example, in numerous B-cell malignancies, CD19 is a primary target since this is highly expressed throughout all stages of B-cell development and differentiation (101). CAR T-cell therapy has shown great efficacy in treating DLBCL, FL, MCL, B-cell ALL, and multiple myeloma. In fact, studies have corroborated CAR T-cell therapy efficacy and toxicity with standard of care products in real-world investigations (102). Additionally, CAR T-cells are FDA-approved in multiple lymphoproliferative disorders including DLBCL (103–105), MCL (106–108), and FL (109). **Table 3** summarizes the currently available CAR T-cell therapies and their FDA-approved clinical indications as of July 31, 2022. Seeing as how effective CAR T-cell therapy has been in the R/R setting for lymphoproliferative disorders, studies are currently being conducted to investigate incorporating CAR T-cell therapy in earlier lines of therapy (110, 111). The main toxicities associated with CAR T-cell therapy are cytokine release syndrome (CRS) and immune effector cell-associated neurotoxicity syndrome (ICANS). Studies have shown that certain therapies utilized prior to CAR T-cell therapy – including bridging and prophylaxis – may influence toxicity profiles and outcomes, hence we need to choose prior therapies carefully (112–116). To minimize the occurrences of CRS and ICANS, studies are investigating combinatorial approaches with the hope that these approaches could potentially be used to decrease toxicity and increase efficacy (117). As a cautionary note, not all combinations will serve as effective therapies; for example efficacy outcomes and peak CAR T-cell levels seem to be similar between patients treated with axicabtagene ciloleucel plus atezolizumab (an immune checkpoint inhibitor) as part of the ZUMA-6 trial compared to historical outcomes as part of the ZUMA-1 trial for axicabtagene ciloleucel alone (118). These malignancies have developed resistance to therapy through alteration of the CD19 marker itself through mechanisms such as frameshift mutations leading to nonsense mutation mediated CD19 decay (81). Other studies found that resistance to anti-CD20 CAR T-cell therapy arose from the tumor cells downregulating the expression of CD20 (69). In B-cell ALL, studies found that the tumor cells switch from B-cell lineage to myeloid lineage after CAR T-cell therapy through a mixed-lineage leukemia (*MLL*) gene rearrangement on chromosome 11q23 (82). Finally, in almost all B-cell malignancies, studies have identified T-cell exhaustion as a contributing factor to the poor persistence of CAR T-cells after infusion. Furthermore, studies have found that enhanced PD-L1 pathway signaling directly contributes to T-cell exhaustion (83). In fact, increased PD-L1 signaling downregulates CD28 co-domain signaling – a signal that is essential for the proper activation of CAR T-cells after the CAR molecule binds to the antigen on tumor cells (83). Thus, PD-L1 interferes with the proliferation and cytotoxicity of T-cells, conferring resistance to therapy (83). Strategies to overcome resistance are being developed and studied – including the addition of small molecules and monoclonal antibodies (102).

**Table 3:** Summary of PI3K inhibitors, their clinical indications, and FDA status as of July 31, 2022.

Agent	Target	Isoform IC_50_				Clinical indication	FDA status	Black box warnings
		PI3K alpha	PI3K beta	PI3K gamma	PI3K delta			
Idelalisib	PI3K delta	820	565	89	2.5	FL and SLL	Approved January 2014; Withdrawn January 2022	Fatal and serious toxicities: hepatic, severe diarrhea, colitis, pneumonitis, and intestinal perforation
Copanlisib	PI3K alpha and delta	0.5	3.7	6.4	0.7	3L FL	Approved June 2021	None
Umbralisib	PI3K delta and casein kinase CK1-epsilon	>1000	1116	1065	22	2L MZL and 4L FL	Approved February 2021; Withdrawn June 2022	Not applicable
Duvelisib	PI3K delta and gamma	1602	85	27	2.5	CLL and SLL	Approved September 2018	Fatal and serious toxicities: infections, diarrhea, colitis, cutaneous reactions, and pneumonitis

*Parsaclisib is a PI3K delta inhibitor which was being explored in clinical trials for 3L FL; nevertheless, its application was withdrawn in January 2022. Zandelisib is a PI3K delta inhibitor that is currently still being explored in clinical trials for 3L FL at the time of this publication.

*IC_50_, half maximal inhibitory concentration; PI3K, phosphatidylinositol-3-kinase; CK1, casein kinase.

## Conclusion

**Figure 1:**
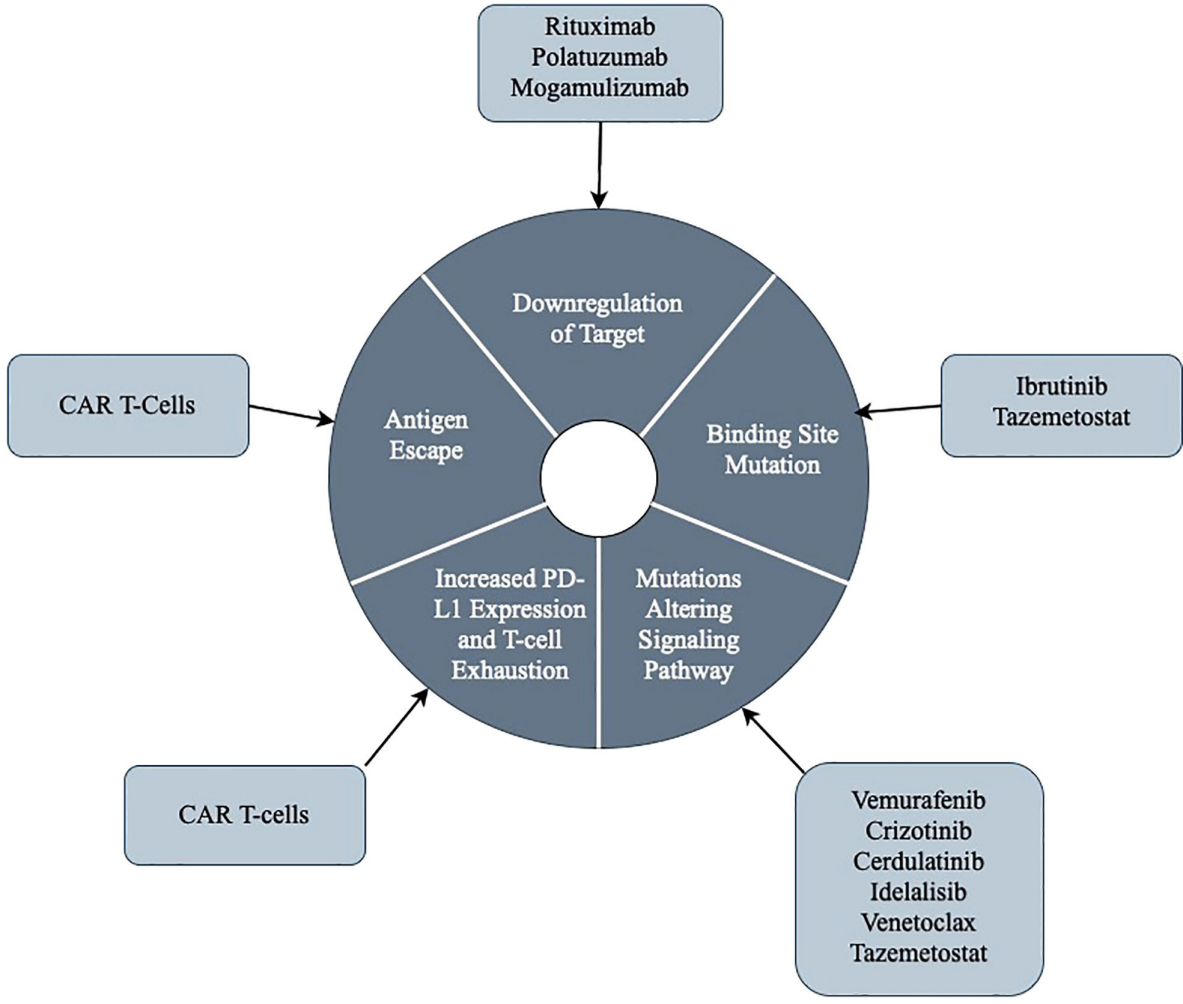
An illustrative summary of the general themes regarding mechanisms of resistance to targeted therapies.

Targeted therapies in lymphoproliferative disorders have made great breakthroughs in treating aggressive malignancies. However, tumor cells continually develop new strategies for survival, and thus mechanisms of resistance to even the most specific agents. We have discussed the currently understood mechanisms of resistance to the most utilized targeted agents in lymphoproliferative diseases, and this has been summarized in **Table 4**. We also have discussed the general common themes regarding mechanisms of resistance to targeted agents, and we illustrated this in **Figure 1**. We eagerly await further studies that identify methods to re-sensitize tumor cells to treatment to increase response rates.

**Table 4:** Summary table of the currently available CAR T-cell therapies and their FDA-approved clinical indications as of July 31, 2022.

Indication	Tisagenlecleucel	Axicabtagene ciloleucel	Brexucabtagene autoleucel	Lisocabtagene maraleucel	Idecabtagene vicleucel	Citacabtagene autoleucel
R/R/ DLBCL	Yes	Yes	No	Yes	No	No
R/R/ High-Grade B-cell Lymphoma	Yes	Yes	No	Yes	No	No
R/R Primary Mediastinal B-cell Lymphoma	No	Yes	No	Yes	No	No
R/R DLBCL Arising from Follicular Lymphoma	Yes	Yes	No	Yes	No	No
R/R/ DLBCL Arising from Indolent Lymphoma	No	No	No	Yes	No	No
R/R Follicular Lymphoma G1-3A	Yes	Yes	No	No	No	No
R/R Follicular Lymphoma G3B	Yes	Yes	No	Yes	No	No
R/R Mantle Cell Lymphoma	No	No	Yes	No	No	No
R/R B-cell precursor acute lymphoblastic leukemia	Yes*	No	No	No	No	No

*Up to age 25 years.

## Author contributions

AD and JM contributed to the writing of the manuscript. All authors contributed to the article and approved the submitted version.

## Conflict of interest

JM: consulting – Pharmacyclics/Abbvie, Bayer, Gilead/Kite Pharma, Pfizer, Janssen, Juno/Celgene, BMS, Kyowa, Alexion, Fosunkite, Innovent, Seattle Genetics, Debiopharm, Karyopharm, Genmab, ADC Therapeutics, Epizyme, Beigene, Servier, Novartis, Morphosys/Incyte, Mei pharma, Zodiac; research funding – Bayer, Gilead/Kite Pharma, Celgene, Merck, Portola, Incyte, Genentech, Pharmacyclics, Seattle Genetics, Janssen, Millennium. Honoraria from Targeted Oncology, OncView, Curio, Kyowa, Physicians’ Education Resource, Dava, Global clinical insights, MJH, Shanghai Youyao, and Seattle Genetics; speaker’s bureau – Gilead/Kite Pharma, Kyowa, Bayer, Pharmacyclics/Janssen, Seattle Genetics, Acrotech/Aurobindo, Beigene, Verastem, AstraZeneca, Celgene/BMS, Genentech/Roche.

The remaining author declares that the research was conducted in the absence of any commercial or financial relationships that could be construed as a potential conflict of interest.

## Publisher’s note

All claims expressed in this article are solely those of the authors and do not necessarily represent those of their affiliated organizations, or those of the publisher, the editors and the reviewers. Any product that may be evaluated in this article, or claim that may be made by its manufacturer, is not guaranteed or endorsed by the publisher.
